# Functions, mechanisms, and therapeutic implications of METTL14 in human cancer

**DOI:** 10.1186/s13045-022-01231-5

**Published:** 2022-02-03

**Authors:** Qian Guan, Huiran Lin, Lei Miao, Huiqin Guo, Yongping Chen, Zhenjian Zhuo, Jing He

**Affiliations:** 1 Department of Pediatric Surgery, Guangzhou Institute of Pediatrics, Guangdong Provincial Key Laboratory of Research in Structural Birth Defect Disease, Guangzhou Women and Children’s Medical Center, Guangzhou Medical University, 9 Jinsui Road, Guangzhou, 510623 Guangdong China; 2 School of Medicine, South China University of Technology, Guangzhou, 510006 Guangdong China; 3 Faculty of Medicine, Macau University of Science and Technology, Macau, 999078 China; 4 Laboratory Animal Center, School of Chemical Biology and Biotechnology, Peking University Shenzhen Graduate School, Shenzhen, 518055 China

**Keywords:** RNA modification, m^6^A, METTL14, Cancer, Drug discovery

## Abstract

RNA modification plays a crucial role in many biological functions, and its abnormal regulation is associated with the progression of cancer. Among them, N^6^-methyladenine (m^6^A) is the most abundant RNA modification. Methyltransferase-like 14 (METTL14) is the central component of the m^6^A methylated transferase complex, which is involved in the dynamic reversible process of m^6^A modification. *METTL14* acts as both an oncogene and tumor suppressor gene to regulate the occurrence and development of various cancers. The abnormal m^6^A level induced by METTL14 is related to tumorigenesis, proliferation, metastasis, and invasion. To date, the molecular mechanism of METTL14 in various malignant tumors has not been fully studied. In this paper, we systematically summarize the latest research progress on METTL14 as a new biomarker for cancer diagnosis and its biological function in human tumors and discuss its potential clinical application. This study aims to provide new ideas for targeted therapy and improved prognoses in cancer.

## Introduction

Among posttranscriptional modifications, more than 100 different types of RNA chemical modifications have been identified. N^6^-methyladenosine (m^6^A) modifications account for approximately 50% of all methylated RNA [1] and are one of the most common and abundant internal modifications [2, 3]. It is found in almost all eukaryotes and in some bacteria, viruses, yeasts, and plants [4, 5]. In 1974, the presence of a methyl substituent at N-6 of adenosine in nucleic acids was first identified in purified poly (A) RNA fragments [6, 7]. Subsequent studies confirmed that it is mainly present in the RRACH motif (where R = A/G, H = A/C/U) and is enriched in the 3' untranslated regions (UTRs), near the stop codon, and in the internal exon.

m^6^A methylation is widely present in mRNA, miRNAs, and long noncoding RNAs [8–11] and is involved in the basic pathophysiological metabolic processes of RNA, including splicing, nuclear output, translation, decay, folding, and RNA–protein interactions [12–15]. This newly identified type of modification plays an important role in regulating gene expression, which has become known as RNA epigenetics. In human physiology, m^6^A methylation plays a critical role in embryonic stem cell differentiation, meiosis, DNA repair, circadian rhythm, tissue development, and tumorigenesis [16–19]. Abnormalities in m^6^A methylation result in embryonic development disorders, failure of differentiation, neurological diseases, and tumorigenesis [20–23].

**Fig. 1:**
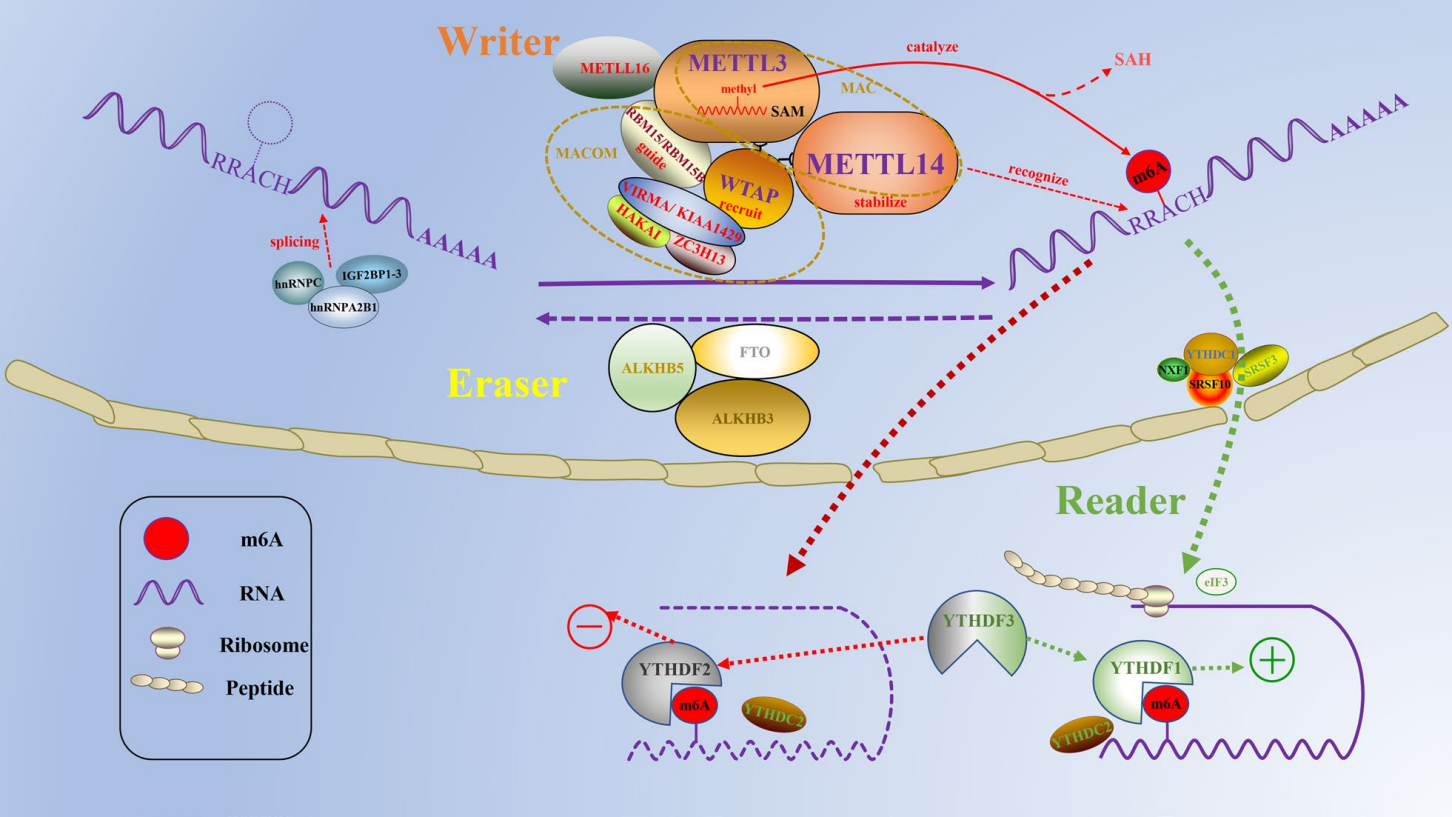
Molecular composition of the m^6^A RNA methylation. m^6^A is installed by “writers” (METTL3/14, WTAP, RBM15/15B, VIRMA, HAKAI, ZC3H13, METTL5/16, and ZCCHC4), removed by “erasers” (FTO, ALKBH5, and ALKBH3), and recognized by “readers” (YTHDC1/2, YTHDF1/2/3, IGF2BP1/2/3, HNRNPA2B1/C/G, and eIF3)

Functionally, m^6^A is divided into “writer” [24, 25], “eraser” [26], and “reader” [27] (Table 1, Fig. 1). m^6^A modification is a dynamically reversible pathway that mainly relies on erasers to encode m^6^A demethylase and remove m^6^A modifications in RNA molecules. At present, the known erasing genes include FTO and ALKBH5 [28]. FTO can affect the splicing and stability of mRNA by regulating m^6^A modification [29, 30], while ALKBH5-mediated demethylation affects the output and metabolism of mRNA and the assembly of mRNA processing factors in nuclear spots [31]. Both of these genes have dual regulatory effects on the occurrence and development of tumors. They catalyze the conversion of m^6^A to N6-hydroxymethyladenosine (hm^6^A) and hm^6^A to N6-formyladenosine (f6A) in two steps. Once formed, f6A spontaneously transforms into adenosine (A) [32]. In recent years, another demethylation transferase, AlkB homolog 3 (ALKBH3), has been identified, which may have a similar process [33, 34]. The m^6^A “reader” is responsible for identifying m^6^A methylated transcripts and generating functional signals, including the YTH domain family of proteins (YTHDC1/2, YTHDF1/2/3) [35–39], IGF2 mRNA binding protein (IGF2BP1/2/3) [40, 41], eukaryotic initiation factor 3 (eIF3) [42], and the heterokaryotic nuclear RNA protein family (HNRNPC, HNRNPG) [43–45]. These readers have been shown to mediate RNA splicing, nuclear export, translation efficiency, RNA stability, and RNA decay [37]. Different reading proteins recognize different m^6^A sites and perform different functions [46]. For example, YTHDF1/YTHDF3 identifies the m^6^A sequence in the ITGA6 3' UTR and promotes the translation of target genes that affect the malignant progression of bladder cancer [47]. YTHDF2 recognizes the methylation of SOCS2 and promotes its mRNA degradation to further regulate HCC cell proliferation and migration [48]. Methylation of RNA molecules is catalyzed by the methyltransferase complex (MTC), known as the "writer" protein, which consists of METTL3, METTL14, Wilms tumor 1 associated protein (WTAP), RNA-binding motif protein 15/15B (RBM15/15B), zinc finger CCCH-type containing 13 (ZC3H13) proteins, Vir-like m^6^A methyltransferase-associated (VIRMA/KIAA1429), and Cbl proto-oncogene like1 (CBLL1/Hakai) [49–53]. METTL3 is the only methyltransferase with catalytic activity, but it needs to bind to METTL14 to be effective [54]. The two combine to form a core complex (also known as the m^6^A-METTL complex, MAC) that catalyzes m^6^A methylation of most RNA. Other components of MTC form regulatory complexes (also known as m^6^A-METTL associated complexes, MACOM) that direct the core complex to specific site regions of RNA and provide binding sites, leading to increased catalytic activity [50, 55]. In addition, several studies have demonstrated the novel readers METTL16, METTL5, and ZCCHC4, which mediate m^6^A modifications of U6 snRNA, 18S rRNA, and 28S rRNA, respectively [56–62]. "Writers" and "erasers" can effectively install and remove mRNA methylation, and they work together to achieve a stable, dynamically balanced reversible process [63]. After the completion of the methylated splicing modification, the mature mRNA will undergo nuclear export and recognition by the reading protein, leading to further functional realization [64, 65].

**Table 1:** The functions of m^6^A enzymes in RNA metabolism

Type	Factors	Function
Writers	METTL3	Catalyzes m^6^A modification [52]
	METTL14	Stabilizes the structure of MTC and recognizes target RNAs [68]
	WTAP	Contributes to the localization of METTL3-METTL14 heterodimer to the nuclear speckle [49, 70]
	VIRMA	Recruits the MTC to the special RNA site and interacts with polyadenylation cleavage factors CPSF5 and CPSF6 [51]
	BM15/15B	Recruits METTL3-METTL14 heterodimer to target transcripts [53]
	ZC3H13	Bridges WTAP to the mRNA-binding factor Nito [55]
	ZCCHC4	Responsible for m^6^A modification of 28S rRNA [60–62]
	METTL16	Catalyzes m^6^A modification in U6-snRNA and participates in pre-RNA splicing [56, 57]
	METTL5	Responsible for m^6^A modification of 18S rRNA [58, 59]
Erasers	FTO	Removes m^6^A modification [29–31]
	ALKBH5	Removes m^6^A modification [31, 58, 110]
	ALKBH3	Removes m^6^A modification [33, 34]
Readers	YTHDC1	Impacts mRNA splicing and nuclear export [37]
	YTHDC2	Promotes RNA decay and translation [38]
	YTHDF1	Enhances the translational rates of its mRNA targets [35]
	YTHDF2	Induces mRNA degradation [36]
	YTHDF3	Promotes mRNA translation (YTHDF1) and decay (YTHDF2) [39]
	IGF2BP1/2/3	Promotes mRNA stability and translation [41]
	HNRNPA2B1/C/G	Regulates primary miRNA processing, mRNA structure and alternative splicing [44, 45]
	eIF3	Promotes mRNA translation [42]

Although METTL14 does not have true catalytic activity, it serves as an important adapter for METTL3 activity to enhance methyltransferase activity by recognizing RNA substrates and methyl localization [66–68]. As an allosteric activator of METTL3 activity, METTL14, as an inactivated methyltransferase and allosteric activator of METTL3 activity, is involved in the development of various tumors. When METTL14 is mutated at cancer-associated sites, this reduces the catalytic activity and substrate specificity of the enzymes involved, leading to the reversal of methylation efficiency of consensus GGACU and non-consensus GGAUU sequences (decreased methylation at consensus sites and increased methylation at non-consensus sites), resulting in the occurrence of cancer [66, 69]. This paper reviews the research progress in understanding the role of METTL14 in the molecular mechanism of various malignant tumors and the biological processes involving METTL14. In addition, we discuss the structure and function of the METTL3-METTL14 heterodimer, the association of METTL14 with histone modification and potential therapeutic strategies for the dysregulation mechanisms of METTL14.

### Structure and function of the METTL3-METTL14 heterodimer

**Fig. 2:**
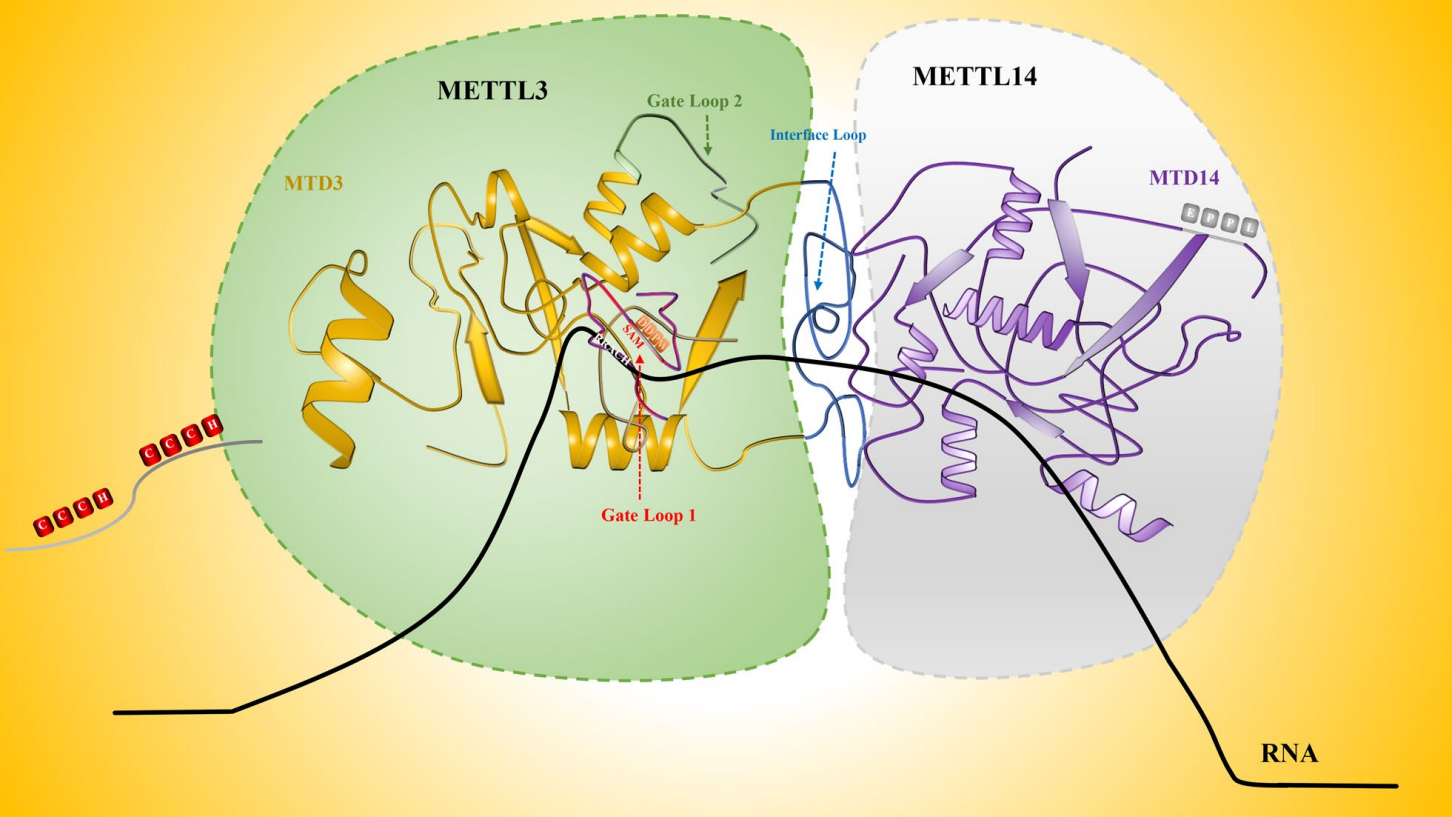
The METTL3-MEETL14 methyltransferase complex. MTD3 of METTL3 is the real catalyst, while METTL14 stabilizes the structure and promotes RNA substrate recognition to improve methylation transferase activity. The catalytic cavity of METTL3 (DPPW motif) has an open conformation and binds to a cofactor (SAM), and METTL14 (EPPL motif) assumes a closed conformation. The two CCCH (ZnF) moieties of METTL3 are required for RNA substrate binding

METTL3 and METTL14 are essential components of the methyltransferases complex, which form a stable heterodimer in a 1:1 ratio [69]. Both of them contain the methyltransferase domain (MTD) [70]. MTD3 is comprised of 357–580 AA residues, which includes three loops (gate loop 1, gate loop 2, and interface loop), two CCCH motifs, catalytic sites (DPPW motif), and S-adenosylmethionine (SAM) binding sites [54, 70, 71]. Among these, SAM binding sites are mainly contained in loop 1 and loop 2, while the remaining rings, namely, the interface rings, have a large area and extensive contact with the METTL14 MTD [54]. METTL3 can transfer SAM methyl groups to the adenine base of RNA to produce homocysteine (SAH) to achieve methyl transfer [70]. In addition, its catalytic cavity has only the conservative motif EPPL [70]. Although MTD14 is structurally similar to MTD3, it lacks SAM binding sites, and thus it does not have catalytic activity [71]. However, METTL3 alone has weak catalytic activity, which is greatly increased only when combined with METTL14 [72]. Some studies have explained this phenomenon, suggesting that METTL14 provides an RNA-binding scaffold that plays an important role in maintaining the structural integrity of binary complexes and recognizing RNA substrates [73]. It is worth noting that some studies have reported that the C-terminal RGG domain of METTL14 contributes to its recognition function [74]. However, these studies are not sufficient. We still do not understand the mechanism by which this structure helps to identify targets, and whether there are other structures that help in this identification. If there are, it is of interest to know what are they and how they interact. In addition, it has been found in recent years that the METTL3-METTL14 complex has a certain repair effect on DNA damaged regions in vitro [75]. Under the same conditions, the METTL3-METTL14 heterodimer ssDNA methylation rate is much higher than that of ssRNA, and the single-stranded DNA has catalytic activity, while the double-stranded DNA does not [76]. This result provides new knowledge about the METTL3-METTL14 complex (Fig. 2).

### *METTL14* functions as an antioncogene

**Fig. 3:**
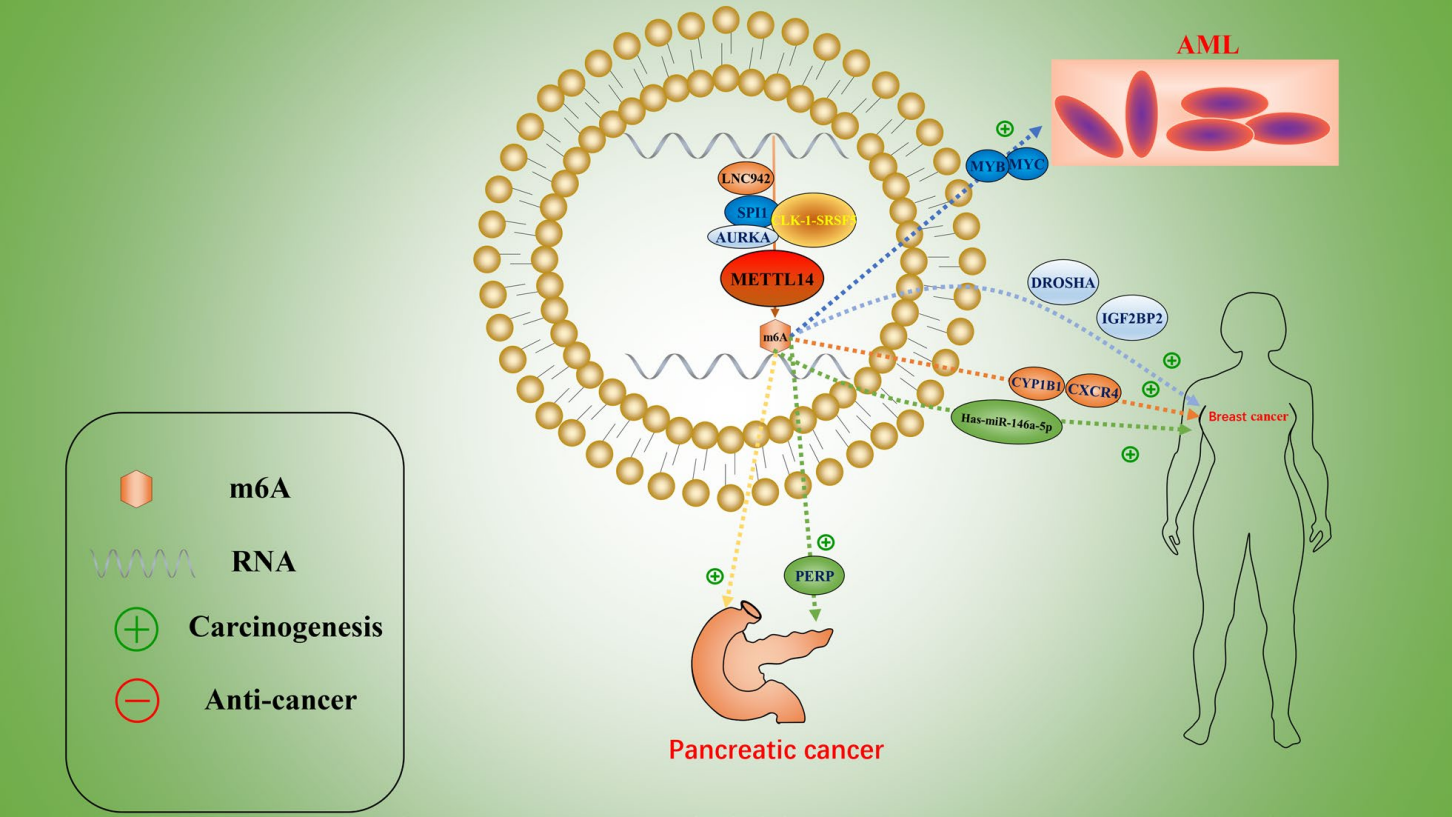
The stimulatory role of METTL14 in human cancers, including AML, breast cancer, and pancreatic cancer

In most tumors, *METTL14* acts as an antioncogene, downregulating the level of m^6^A in tumor cells by exerting its function as a m^6^A methyltransferase to suppress the occurrence and progression of tumors (Fig. 3).

### Colorectal cancer

Colorectal cancer (CRC) is a malignant disease with a high incidence worldwide. According to statistics, there are 945,000 new cases and nearly 700,000 deaths every year, making it one of the top four causes of cancer death [77–79]. Liu et al. confirmed that METTL14 expression was upregulated in CRC tissues, and survival analysis showed that the METTL14 expression level was significantly correlated with the prognosis of CRC [80]. Chen et al. further found that downregulation of METTL14 and m^6^A promoted the growth, invasion, and migration of cancer cells. The specific molecular mechanism is that overexpression of METTL14 affects the binding of DGCR8 and primiR-375 and regulates the level of miR-375. Furthermore, it further downregulates Yes-associated protein 1 (YAP1) to inhibit the growth of cancer cells and inhibit the invasion and migration of cancer cells by downregulating SP1 [81]. To better understand how METTL14 inhibits the malignant progression of cancer cells, some studies have also included reading proteins. For example, Wang et al. showed that methyl-CpG binding protein 2 (MeCP2) and METTL14 enhance the expression of Kruppel like factor 4 (KLF4) protein and mRNA in an IGF2BP2-dependent manner and inhibit the proliferation, metastasis, and invasion of CRC [82]. Chen et al. also confirmed that METTL14 mediates epithelial-mesenchymal transformation (EMT) and that PI3K/AKT signal transduction inhibition of CRC cell migration and invasion works partly through SRY-related high-mobility-group box 4 (SOX4). When *METTL14* is knocked down, SOX4 mRNA is increased, and this process depends on YTHDF2 recognition [83]. Another study proved that lncRNA XIST was the downstream target gene of *METTL14* through transcriptomic sequencing (RNA-seq) and methylated RNA immunoprecipitation (Me-RIP). When *METTL14* was knocked down, the m^6^A level of XIST was downregulated and mRNA expression increased, thus promoting the malignant progression of CRC. In addition, METTL14 downregulates XIST-dependent m^6^A-YTHDF2 pathways [84]. Significant downregulation of METTL14 and YTHDC2 may be a potential prognostic biomarker for rectal cancer [85]. Moreover, Dong et al. revealed that in tumor-associated macrophages (TAMs) of CRC, knockout of METTL14 results in a decrease in m^6^A levels, an increase in EBI3, and dysfunction of antitumor T cells, which then promots the malignant progression of tumors [86]. In conclusion, studies have shown that the relationship between *METTL14* and CRC is relatively extensive and involves the immune microenvironment. It is worth mentioning that studies that examine the *METTL14* gene in the context of the immune system represent an interesting direction.

### Liver cancer

Hepatocellular carcinoma (HCC) is a highly malignant tumor with high recurrence and metastasis rates and poor mortality. It is the most common fatal malignant tumor worldwide [87–89]. Although the risk indicators for HCC are well understood, the underlying molecular mechanisms remain unclear. Traditionally, it is believed that the occurrence of liver cancer is related to chromosome gain/loss and somatic mutation. In recent years, increasing evidence has shown that epigenetics plays a vital role in regulating the occurrence of liver cancer [90]. Shi et al. showed that *METTL14* gene expression was significantly downregulated in HCC, which was associated with a poor prognosis in cancer patients. EGFR was identified as the downstream target gene of METTL14 by RNA sequencing and m^6^A sequencing, and METTL14 was shown to regulate the EGFR/PI3K/AKT signaling pathway in an m^6^A methylation-dependent manner, thereby inhibiting EMT and invasion of cancer cells [91]. However, in metastatic liver cancer, miR-126 is the target gene of *METL14*, and this pathway downregulates the expression of miR-126 to promote tumor metastasis by regulating the interaction between DGCR8 and primiR-126 [92]. Du et al. found that METTL14-mediated m^6^A modification maintained the stability of USP48 mRNA, thus participating in the regulation of HCC, and revealed that the METTL14-USP48-SIRT6 axis plays an inhibitory role by regulating glycolysis [93]. In addition, Li et al. proposed that METTL14 may inhibit the occurrence of HCC by upregulating the expression levels of cysteine sulfonate decarboxylase (CSAD), glutamic oxalacetic transaminase (GOT2), and cytokine signaling inhibitor 2 (SOCS2) [94]. These studies show that METTL14 has a significant impact on liver cancer.

### Breast cancer

Breast cancer remains a serious challenge for women around the world, with a five-year survival rate of less than 30% for patients with advanced cancer [95]. Further research on the molecular mechanism of breast cancer is becoming increasingly important to improve the survival rate and clinical prognosis. Several studies have demonstrated reduced METTL14 expression in breast cancer tissues [96, 97]. Its expression level was shown to be negatively correlated with tumor grade [98]. The lower the expression level was, the worse the prognosis [97]. Overexpression of METTL14 can lead to a decrease in m^6^A levels and inhibit the migration and proliferation of cancer cells [96].

### Endometrial carcinoma

Endometrial cancer (EC) is a common malignant gynecological tumor worldwide. Even in early-stage cancer, routine surgery has a great impact on the fertility of patients, and the development of effective interventions is of great importance [99, 100]. Ma et al. reported that m^6^A RNA methylation was closely associated with the clinicopathological stage and prognosis of endometrial cancer and that METTL14 was used as a potential marker for the diagnosis and prognosis of endometrial cancer [101]. Liu et al. found that the R298P mutation in the key component of METTL14 leads to a reduction in m^6^A methylation and activation of the AKT pathway, thereby promoting the proliferation and migration of endometrial cancer cells. The increase in AKT activity depended on the decrease in PHLPP2 expression and the increase in mTORC2 expression [102].

### Bladder cancer

Bladder cancer (BC) is the most universal tumor of the urinary system and has become the fifth most common cancer in the United States, producing an estimated 81,400 cases in 2019 [22, 103]. Gu et al. found that low expression of METT14 in BC and bladder tumor-initiating cells (TICs), decreased m^6^A levels, and m^6^A levels were associated with clinical severity and prognosis. Knockout of METTL14 enhances Notch1 expression and stability, promoting the development of BC and bladder TIC self-renewal [104]. The METTL14-m^6^A-Notch1 pathway plays a critical role in bladder tumorigenesis and bladder TICs. Zhang et al. revealed that isorhapontigenin (ISO) inhibited the migration, invasion, and EMT of BC cells by upregulating METTL14 mRNA expression and decreasing vimentin protein levels by activating the transcription factor FOXO3a [105].

### Neuroblastoma

Neuroblastoma (NB) is the most common tumor in infants and young children. It originates from the sympathetic ganglion and bilateral adrenal glands and has the highest morbidity and mortality in infancy [106, 107]. Wang et al. proposed that METTL14 combined with WTAP, HNRNPC, YTHDF1, and IGF2BP2 contributed to the prognosis of NB and could be used as new targets for clinical treatment [107]. Our group first found that some SNPs in the *METTL14* gene were closely associated with the risk of neuroblastoma. *METTL14* gene rs298982 G > A and rs62328061 A > G were significantly associated with reduced susceptibility to neuroblastoma, while rs9884978 G > A and rs4834698 T > C were associated with increased susceptibility to neuroblastoma [108].

### Glioblastoma

Glioblastoma, which is the most common primary brain tumor, involves self-renewing glioblastoma stem cells (GSCs). The high mortality rate of glioblastoma is largely due to the tumor heterogeneity and therapeutic resistance of GSCs [109, 110]. Studies have shown that *METTL14* gene knockout significantly promotes the generation and development of GSCs, possibly by affecting the enrichment of ADAM19 m^6^A to promote the expression of ADAM19, resulting in the self-renewal and tumorigenesis of GSCs [111].

### Kidney cancer

Kidney cancer, also known as renal cell carcinoma (RCC), is a malignant tumor with the highest mortality rate in the genitourinary system, among which the most common pathological type is clear cell carcinoma of the kidney (ccRCC) [112]. According to statistics, the United States reports 73,820 cases a year and an estimated 14,770 deaths [113]. Compared with normal kidney tissue, the *METTL14* mRNA level was significantly reduced in ccRCC [114]. Gong et al. confirmed that knockdown of METTL14 reduced m^6^A levels and increased mRNA and protein levels of P2RX6, which then promoted the migration and invasion of RCC through the ATP-P2RX6-Ca^2+^-P-ERK1/2-MMP9 signaling pathway [115]. Liu et al. found that METTL14 inhibited the proliferation and migration of renal carcinoma by inhibiting the expression of long noncoding RNA nuclear enriched abundant transcript 1_1 (NEAT1_1) by YTHDF2 [116]. METTL14 may be an independent prognostic indicator of RCC and ccRCC in univariate and multivariate Cox regression analyses [117–120]. The reduced METTL14 expression predicts a poor prognosis of the tumor. Studies have suggested that in RCC, the miRNA/mRNA-hsa-miR-1307- 3p/METTL14 pathway may regulate the occurrence and development of tumors and play an important role in clinical applications [121]. In addition, some studies have found that METTL14 is positively correlated with PTEN [114], which indicates that METTL14 plays an inhibitory role in RCC by regulating PTEN. Notably, Zhang et al. revealed that knockdown down of *METTL14* enhances the stability of bromodomain PHD finger transcription factor (BPTF) mRNA and activates downstream targets such as enolase 2 and SRC proto-oncogene nonreceptor tyrosine kinases, leading to glycolytic reprogramming that drives RCC metastasis [122]. This provides a mechanism for the synergistic effect of m^6^A modification and glycolysis.

### Papillary thyroid carcinoma

The most common type of thyroid cancer is papillary thyroid carcinoma (PTC), with an incidence of more than 80% and a 5-year survival rate of more than 97% with a good prognosis [123]. Zhang et al. demonstrated through RIP and RNA pull-down analysis that lncRNA OIP5-AS1 is a gene downstream of *METTL14*, and that the overexpression of METTL14 regulates MEK/ERK and EGFR pathways through OIP5-AS1/miR-98/ADAMTS8, thus promoting the malignant behavior of PTC cells [124].

### Leukemia

Acute myeloid leukemia (AML) is a common and deadly tumor of the blood system [125]. Alterations in m^6^A levels can affect cell fate and differentiation status [126]. Sun et al. found that METTL14 levels were decreased in E/R positive patients compared with the control group, and it was speculated that the downregulation affected m^6^A modification in related cancer cells, thereby promoting the occurrence of AML [127]. A five-center case–control study found that *METTL14* gene rs298982 G/A and rs1064034 T/A were significantly associated with a reduced risk of ALL in children [128]. METTL14 may be a potential biomarker for the prognosis of ALL.

### Other cancers

In addition to the abovementioned tumors, METTL14 also acts as a tumor suppressor in other tumors. In lung adenocarcinoma (LUAD), Wang et al. found that METTL14 enhanced the stability of human leukocyte antigen complex group11 (HCG11) mRNA and inhibited the growth of lung adenocarcinoma via IGF2BP2/LATS1 [129]. The characteristic expression of the m^6^A regulatory factor in castration-resistant prostate cancer (CRPC) and prostate cancer (PCa) was analyzed. METTL14 was downregulated and correlated with lymph node metastasis of CRPC and was negatively correlated with the Gleason grade in PCa [130]. METTL14 was downregulated in triple-negative breast cancer (TNBC) [118], esophageal cancer (EC) [131], gastric cancer [132], osteosarcoma (OS) [133],Wilms tumor, [134] and oral squamous cell carcinoma (OSCC) [135], and low METTL14 expression was related to poor prognosis. Notably, it has been confirmed that METTL14 knockout can activate Wnt and PI3K-Akt signals to promote the growth and invasion of gastric cancer cells [132]. Of course, these findings of MTTL14 need to be confirmed by subsequent studies.

### *METTL14* acts as an oncogene

**Fig. 4:**
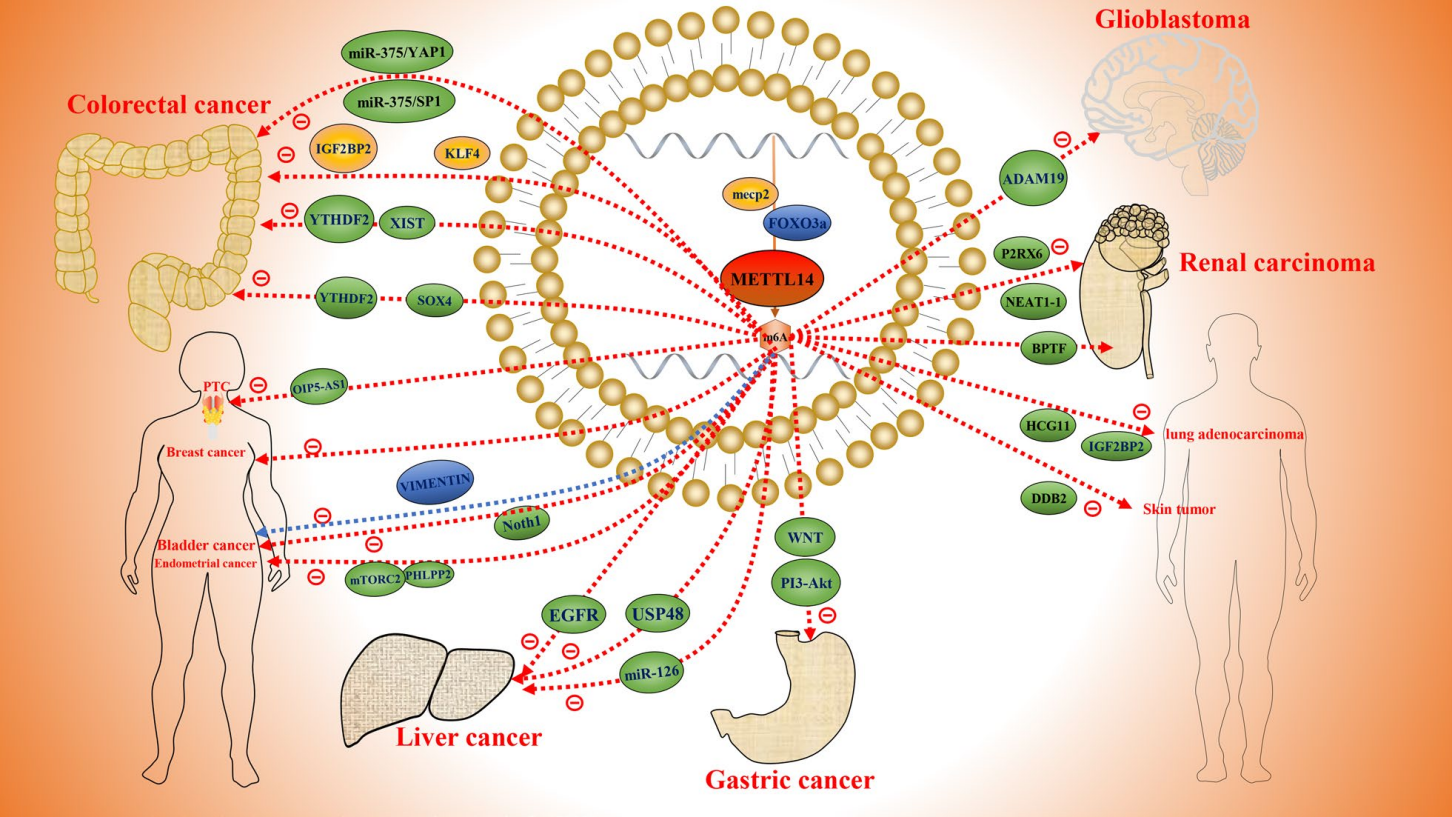
The suppressive role of METTL14 in human cancers, including colorectal cancer, liver cancer, endometrial cancer, bladder cancer, glioblastoma, renal carcinoma, PTC, gastric cancer, breast cancer, skin tumor, and lung adenocarcinoma

Although many studies have shown an inhibitory effect on cancer, METTL14 has also been shown to stimulate the development and progression of tumors in some cases (Table 2). Wang et al. reported that m^6^A levels were elevated in most pancreatic cancer samples and that METTL14 expression was significantly associated with survival. METTL14 overexpression reduces PERP mRNA and protein levels and promotes tumor cell migration and colony formation [136]. The CLK1-SRSF5 axis promotes the proliferation, migration, invasion, and colony formation of pancreatic cancer cells by inhibiting METTL14^△Exon10+^ exon skipping and increasing the m^6^A level [137]. *METTL14* overexpression regulates the expression of hsa-miR-146a-5p through m^6^A modification, thereby promoting breast cancer invasion and migration [138]. Sun et al. found that *LINC00942*, as an oncogene in the occurrence and development of BRCA tumors, promotes METTL14-mediated m^6^A modification and regulates the mRNA stability and protein expression of the downstream genes CXCR4 and CYP1B1, thus promoting tumor growth and development. A new LINC00942-METTL14-CXCR4/CYP1B1 pathway was verified, providing a new approach for the diagnosis and treatment of BRCA [139]. Subsequently, Peng et al. found that the oncogene AURKA enhances the stability of DROSHA mRNA and promotes the oncogenic properties of the DROSHA-STC1 axis by inhibiting the ubiquitination-mediated degradation of the METTL14 protein and improving the recognition ability of IGF2BP2, leading to the malignant progression of breast cancer [140]. Therefore, it is reasonable to speculate that the upregulation of m^6^A in peripheral blood may be a new biomarker for breast cancer and that the upregulation of METTL14 has a better diagnostic role in peripheral blood BC screening [141]. Similarly, Zhao et al. investigated whether m^6^A RNA methylation-related proteins can effectively predict the prognosis of head and neck squamous cell carcinoma (HNSCC). The results showed that the upregulation of METTL14 and WTAP may have a certain guiding significance for prognosis prediction [142]. In addition, Weng et al. reported that METTL14 blocked bone marrow differentiation and promoted cell proliferation in normal hematopoietic stem/progenitor cells (HSPCs) and AML. This occurred because METTL14-mediated m^6^A modification improves the mRNA stability and translation of the downstream target gene MYC/MYB, whereas METTL14 is negatively regulated by SP1. In other words, when SPI1 expression is inhibited, METTL14 upregulates MYC/MYB expression, leading to blocked bone marrow differentiation and cancer [18]. In addition, Martin et al. demonstrated that lowering METTL14 and METTL3 levels promoted bone marrow differentiation [143]. The carcinogenic role of METTL14 in leukemia was emphasized (Fig. 4).

**Table 2:** The function of METTL14 as an m^6^A methyltransferase in human cancer

Role	Cancer type	Upstream	Targets	Reader	Cellular function
Tumor suppressor	Colorectal cancer Colorectal cancer	MeCP2	miR-375/YAP1 miR-375/SP1 KLF4	IGF2BP2	Growth, migration, and invasion [81] Proliferation, invasion, and metastasis [82]
	Colorectal cancer		SOX4	YTHDF2	Invasion and metastasis [83]
	Colorectal cancer		XIST	YTHDF2	Proliferation and metastasis [84]
	Liver cancer		EGFR		Migration, invasion, and EMT [91]
	Liver cancer Liver cancer		miR-126 USP48		Invasion and metastasis [92] Tumorigenesis [93]
	Breast cancer				Growth and metastasis [96]
	Endometrial cancer		PHLPP2/ mTORC2		Proliferation and tumorigenicity [102]
	Bladder cancer		Notch1		Proliferation, self-renewal metastasis, and tumorigenicity [104]
	Bladder cancer	FOXO3a	Vimentin		Migration, invasion, and EMT [105]
	Glioblastoma		ADAM19		Growth, self-renewal, and tumorigenesis [111]
	RCC RCC RCC		P2RX6 NEAT1_1 BPTF		Migration and invasion [115] Growth and metastasis [116] Metastasis and EMT [122]
	PTC LUAD		OIP5-AS1 HCG11	IGF2BP2	Proliferation, migration, and invasion [124] Growth [129]
	Gastric cancer		Wnt/PI3K-AKT		Proliferation and invasion [132]
Oncogene	Skin tumor		DDB2		Autophagy [159]
	Pancreatic cancer		PERP		Proliferation and migration [136]
	Pancreatic cancer	CLK-1-SRSF5			Invasion and metastasis [137]
	Breast cancer		hsa-miR-146a-5p		Migration and invasion [138]
	Breast cancer Breast cancer AML	LNC942 AURKA SPI1	CXCR4/ CYP1B1 DROSHA MYB/MYC	IGF2BP2	Proliferation and growth [139] Proliferation [140] Survival and growth [18]

*AML* Acute myeloid leukemia, *PTC* Papillary thyroid carcinoma, *LUAD* Lung adenocarcinoma, *RCC* Renal cell carcinoma

### Interaction of METTL14 with histone modifications

In a study of embryonic neural stem cells (NSCs), Wang et al. found that knockdown of METTL14 significantly increased the level of acetylation of histone H3 at lysine 27 (H3K27ac), trimethylation of histone H3 at lysine 4 (H3K4me3), and trimethylation of histone H3 at lysine 27 (H3K27me3) and reduced the proliferation ability of neural stem cells. It was further verified that METTL14 regulates histone modification by enhancing the stability of H3K27ac CBP/p300 mRNA [144]. The interaction between m^6^A and histone modification was revealed for the first time. Chen et al. reported that lysine-specific histone demethylation 5C (KDM5C) mediates the demethylation of H3K4me3 in the METTL14 promoter in colorectal cancer and inhibits the transcription of METTL14 [82]. On this basis, Wang et al. found that arginine methylation in the C-terminal region of METTL14 promoted the binding of METTL14 to RNA substrates, METTL3-14 methyltransferase activity and METTL14 interaction with RNA polymerase II [145]. Huang et al. demonstrated that H3K36me3 can directly bind to MTC, and that METTL14 recognizes the core region of H3K36me3 and collaborates with RNA polymerase II to induce methylation of new RNA [146, 147]. In conclusion, the above findings reveal the cross-talk between histone modification and m^6^A modification at the level of epigenetic modification, revealing a new gene regulation mechanism and a further understanding of the recognition mechanism of METTL14.

### Potential clinical treatments

**Fig. 5:**
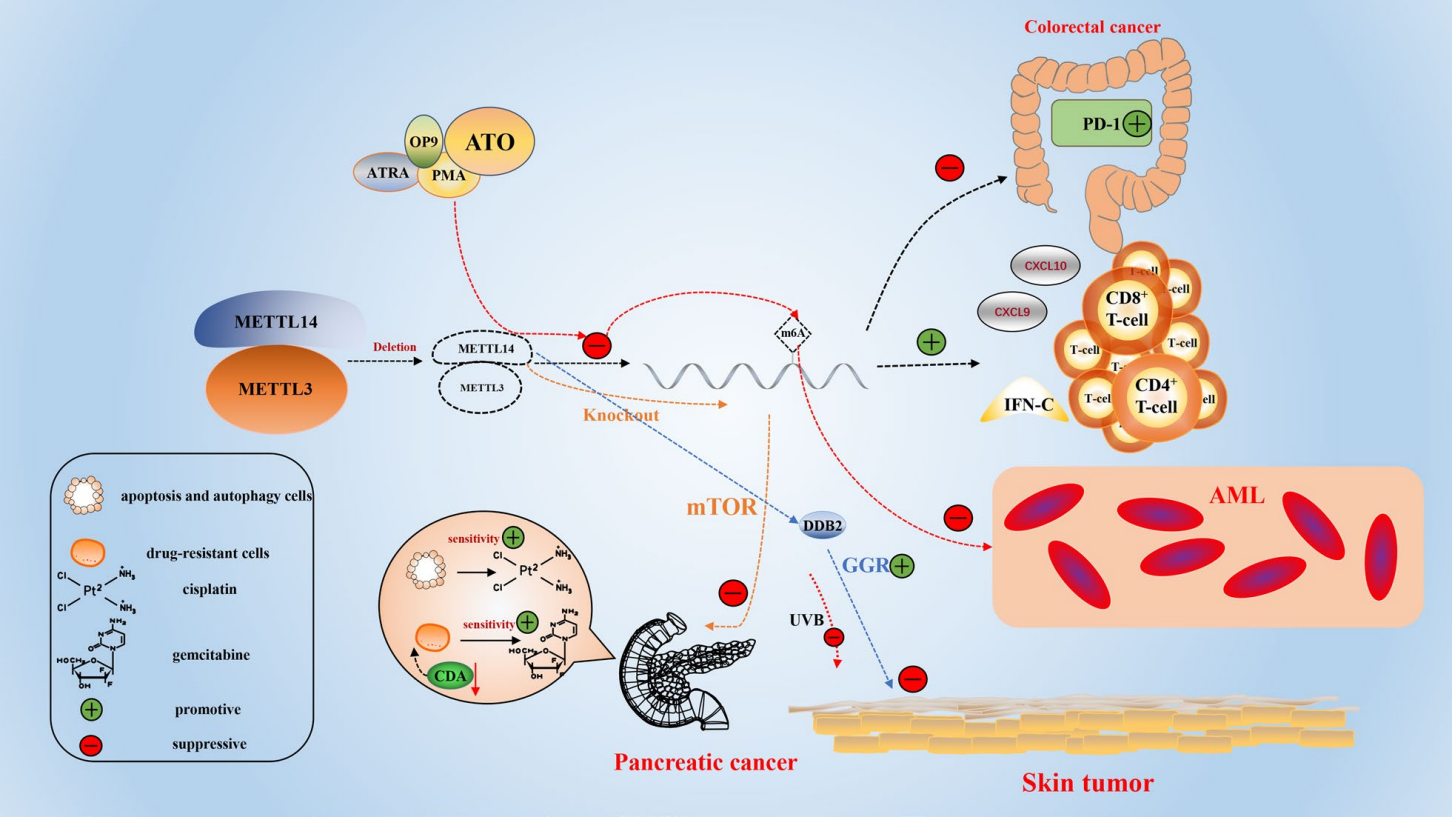
The potential clinical role of METTL14 and METTL3 in certain cancers, including pancreatic cancer, skin tumor, colorectal cancer, and AML

By exploring the relationship between the immune system and tumorigenesis, immunotherapy has become an unprecedented treatment for many cancers [148]. Increased RNA methylation in anticancer immunotherapy affects immune responses [149]. Wang et al. found that inhibition of m^6^A mRNA modification by deletion of METTL14 and METTL3 enhanced the response to programmed cell death-1 (PD-1) therapy in colorectal cancer. The proliferation of CD8^+^T cells and the production of interferon (IFN)-C, CXCL9, and CXCL10 were also induced. It also promotes the accumulation of CD8^+^ and CD4^+^ effector T cells, which inhibit tumor growth, and enhance the efficacy of immunotherapy [150, 151]. In the treatment of AML, all-trans retinoic acid/arsenic trioxide (ATO) [152], differentiation inducers (OP9 medium) [153], PMA [154], and all-trans retinoic acid (ATRA) [155] have been reported to significantly reduce m^6^A levels and the expression of METTL14, thereby promoting myeloid differentiation and inhibiting leukemia growth [156]. In pancreatic cancer, knockout of METTL14 enhances the sensitivity of cancer cells to cisplatin by inducing apoptosis and autophagy through the mTOR signaling pathway [157] and inhibits the expression of cytidine deaminase (CDA), improving the sensitivity of drug-resistant cells to gemcitabine [158]. These studies demonstrate the importance of METTL14 inhibitors in the treatment of tumors. In addition, a recent study found that METTL14 regulates DDB2 translation to promote global genomic repair (GGR) and inhibits ultraviolet B (UVB) radiation to reduce the incidence of skin tumors [159]. Therefore, screening for and designing more effective METTL14 protein inhibitors and activators are expected to provide new anticancer drugs, and targeted therapies in combination with other drugs may become a panacea for controlling many diseases and other forms of cancer (Fig. 5).

## Conclusions and prospects

The occurrence and development of cancer are mainly caused by abnormal genetic changes and epigenetic abnormalities. Abnormal inheritance includes gene mutation, deletion, amplification, and chromosomal translocation [40]. Epigenetics includes DNA, RNA, and histone modifications [160–163]. m^6^A methylation is the most common internal modification of RNA and is of great significance for gene expression regulation [164, 165]. Changes in m^6^A-related genes or proteins affect a variety of biological processes that involve m^6^A methylation, including viral infection [166], stress [167], heat shock [15], DNA damage [168], and the occurrence and development of cancer. It is worth noting that METTL14 can function independently of m^6^A. For example, Liu et al. found that the METTL3-METTL14 complex promotes transcription of the SASP gene and enhances immune surveillance, independent of changes in m^6^A levels [169]. This article reminds us that hotspot proteins should be studied from multiple perspectives with innovative perspectives.

In recent years, with an increasing number of studies on METTL14, some breakthroughs have been made in some aspects, such as mechanisms and pathways of cancer and related metabolic processes, but at the same time, many problems have been exposed. First, METTL14 can recognize the structural support and recognition function of METTL3, but the specific structural basis and molecular mechanism of this recognition and support remain unclear. It is also unclear how METTL14 interacts with other MTC components during tumor development. Second, the METTL3-14 complex has been thought to play a synergistic role. However, studies have shown that METTL3 and METTL14 have opposite regulatory effects on HCC [170]. We hypothesized that METTL3 and METTL14 may have different target preferences and thus trigger different pathway effects. Of course, this requires further experimental verification. In addition, the m^6^A locus of the METTL14 target gene has not been mapped in detail in specific studies of the METTL14 pathway. Finally, it is particularly emphasized that METTL14 has a dual regulatory effect on tumors, and attention should be paid to the use of METTL14 activators or inhibitors to avoid the occurrence of other tumors.

In summary, METTL14 plays an important role in a variety of tumors, regardless of whether they are dependent on m^6^A modification. We look forward to further studies to optimize a targeted METTL14 treatment and enable its use widely in clinical practice.

## Data Availability

Not applicable.

## Abbreviations

METTL14: Methyltransferase-like14
UTR: Untranslated region
MTC: Methyltransferase complex
hm6A: N6-hydroxymethyladenosine
f6A: N6-formyladenosine
ALKBH3: AlkB Homolog 3
IGF2BP: IGF2 mRNA binding protein
eIF3: Eukaryotic initiation factor 3
WTAP: Wilms tumor 1 associated protein
RBM: RNA-binding motif protein
ZC3H13: Zinc finger CCCH-type containing 13
VIRMA: Vir-like m^6^A methyltransferase-associated
CBLL1: Cbl proto-oncogene like1
MAC: M^6^A-METTL Complex
MACOM: M^6^A-METTL associated complexes
MTD: Methylation transferase domain
SAM: S-adenosylmethionine
CRC: Colorectal cancer
YAP: Yes-associated protein1
SOX4: SRY-related high-mobility-group box 4
EMT: Epithelial-mesenchymal transformation
KLF4: Kruppel like factor 4
TAM: Tumor-associated macrophage
HCC: Hepatocellular carcinoma
CSAD: Cysteine sulfonate decarboxylase
GOT2: Glutamic oxalacetic transaminase 2
SOCS2: Cytokine signaling inhibitor 2
AML: Acute myeloid leukemia
EC: Endometrial cancer
BC: Bladder cancer
TICs: Tumor-initiating cells
ISO: Isorhapontigenin
GSCs: Glioblastoma stem cells
RCC: Renal cell carcinoma
PTC: Papillary thyroid carcinoma
PCa: Prostate cancer
NEAT: Nuclear enriched abundant transcript
BPTF: Bromodomain PHD finger transcription factor
LUAD: Lung adenocarcinoma
HCG11: Human leukocyte antigen complex group11
OSCC: Oral squamous cell carcinoma
CRPC: Castration-resistant prostate cancer
TNBC: Triple-negative breast cancer
EC: Esophageal cancer
OS: Osteosarcoma
MeCP2: Methyl CpG-binding protein 2
HNSCC: Head and neck squamous cell carcinoma
KDM5C: Lysine-specific histone demethylation 5C
H3K4me3: H3 lysine 4 trimethylation
PD-1: Programmed cell death-1
ATRA: All-trans retinoic acid
CDA: Cytidine deaminase
UVB: Ultraviolet B
